# Genetic Diversity and Microevolution of *Burkholderia pseudomallei* in the Environment

**DOI:** 10.1371/journal.pntd.0000182

**Published:** 2008-02-20

**Authors:** Narisara Chantratita, Vanaporn Wuthiekanun, Direk Limmathurotsakul, Mongkol Vesaratchavest, Aunchalee Thanwisai, Premjit Amornchai, Sarinna Tumapa, Edward J. Feil, Nicholas P. Day, Sharon J. Peacock

**Affiliations:** 1 Mahidol-Oxford Tropical Medicine Research Unit, Faculty of Tropical Medicine, Mahidol University, Bangkok, Thailand; 2 Department of Biology and Biochemistry, University of Bath, Bath, United Kingdom; 3 Center for Clinical Vaccinology and Tropical Medicine, Nuffield Department of Clinical Medicine, University of Oxford, Churchill Hospital, Oxford, United Kingdom; Charles Darwin University, Australia

## Abstract

**Background:**

The soil dwelling Gram-negative pathogen *Burkholderia pseudomallei* is the cause of melioidosis. The diversity and population structure of this organism in the environment is poorly defined.

**Methods and Findings:**

We undertook a study of *B. pseudomallei* in soil sampled from 100 equally spaced points within 237.5 m^2^ of disused land in northeast Thailand. *B. pseudomallei* was present on direct culture of 77/100 sampling points. Genotyping of 200 primary plate colonies from three independent sampling points was performed using a combination of pulsed field gel electrophoresis (PFGE) and multilocus sequence typing (MLST). Twelve PFGE types and nine sequence types (STs) were identified, the majority of which were present at only a single sampling point. Two sampling points contained four STs and the third point contained three STs. Although the distance between the three sampling points was low (7.6, 7.9, and 13.3 meters, respectively), only two STs were present in more than one sampling point. Each of the three samples was characterized by the localized expansion of a single *B. pseudomallei* clone (corresponding to STs 185, 163, and 93). Comparison of PFGE and MLST results demonstrated that two STs contained strains with variable PFGE banding pattern types, indicating geographic structuring even within a single MLST-defined clone.

**Conclusions:**

We discuss the implications of this extreme structuring of genotype and genotypic frequency in terms of micro-evolutionary dynamics and ecology, and how our results may inform future sampling strategies.

## Author Summary

The soil dwelling Gram-negative bacterium *Burkholderia pseudomallei* is the cause of melioidosis, a serious human infection that occurs in Southeast Asia and northern Australia. The purpose of this study was to evaluate the population genetic structure of *B. pseudomallei* in the environment. To achieve this, we undertook soil sampling and culture for the presence of *B. pseudomallei* in 100 equally spaced points within an area of disused land in northeast Thailand, and undertook detailed genotyping of primary plate colonies isolated from three independent sampling points. Our results demonstrated that multiple *B. pseudomallei* genotypes were present within a single soil sample, and that different genotypes were present at independent but nearby sampling points. The *B. pseudomallei* genetic population was unevenly distributed within a given sample, with a predominant genotype co-existing with several genotypes present as a minority population. We discuss the implications of this structuring of genotypic frequency in terms of micro-evolutionary dynamics and ecology, and how our results may inform future sampling strategies.

## Introduction

The soil dwelling Gram-negative bacterium *Burkholderia pseudomallei* is the cause of melioidosis. This organism is present in the environment across much of southeast Asia and Northern Australia and is increasingly recognised elsewhere, including parts of South America [1],[2]. Infection occurs through bacterial inoculation and contamination of wounds, and more rarely by inhalation and ingestion [1],[3]. Environmental sampling underpins efforts to define the global distribution of *B. pseudomallei* in soil, and the associated geographic distribution of risk to humans and livestock. Sampling is also performed during the investigation of suspected outbreaks, when bacterial genotyping is used to compare *B. pseudomallei* obtained from cases of melioidosis with strains from a specified environment or substance. Environmental sampling would also be required following the deliberate release of *B. pseudomallei* associated with bioterrorist activity. The accuracy of such studies depends on the detection of all of the *B. pseudomallei* genotypes present at a given site with the exception of those present at an extremely low frequency. Informed sampling strategies are also a prerequisite for meaningful comparisons between environmental isolates and those recovered from cases of disease in humans and animals, which provide an important means to identify clones with heightened virulence.

The objective of most published environmental studies of *B. pseudomallei* has been to determine its presence based on culture of soil and/or water in different geographic regions, particularly in Southeast Asia and northern Australia [4]–[17]. Several studies were conducted prior to the initial recognition [18], and later description in 1998 of *B. thailandensis* [19], a putatively non-virulent but closely related species present in the soil which can cause confusion because it has very similar colony morphology characteristics to *B. pseudomallei* on solid media. Yield of *B. pseudomallei* from different soil depths and during different times of the year have also been examined [8],[12], and quantitative culture of *B. pseudomallei* has been performed in several countries [9],[14],[16],[17]. The combined results of these studies indicate that *B. pseudomallei* count in soil varies with sampling depth and calendar period (and associated weather conditions), and varies in presence and quantity both within and between countries. Environmental sampling has also been employed during an investigation of a suspected outbreak of melioidosis in northern Australia in which a case cluster was linked to the water supply through genotyping by pulsed field gel electrophoresis (PFGE) [20], and as part of an environmental sampling program in the same region [21]. Two studies have also compared bacterial genotypes of environmental versus disease-associated strains using ribotyping or multilocus sequence typing (MLST) [22],[23]. The soil sampling methodology used previously to detect and in some cases quantify *B. pseudomallei* using culture methods has varied. The quantity of soil sampled ranged from 3 g to 100 g, although the addition of water to the sample, use of the supernatant for culture, and the ratio of water to soil used are common to many studies. An exception was the identification of *B. pseudomallei* following passage through hamsters inoculated with soil extracts [15].

Despite the importance of environmental sampling for the presence of *B. pseudomallei*, the genetic variability of this organism within a single sample or between adjacent sampling points is not known, and the strategies necessary to ensure sampling of a genetically unbiased *B. pseudomallei* population are undefined. The aim of this study conducted in northeast Thailand was to address these issues. We describe the presence of multiple *B. pseudomallei* genotypes within a single soil sample, and the presence of different *B. pseudomallei* genotypes at independent but nearby sampling points. The *B. pseudomallei* genetic population was unevenly distributed within a given sample, with a predominant genotype co-existing with several genotypes present as a minority population. We discuss the implications of this structuring of genotypic frequency in terms of micro-evolutionary dynamics and ecology, and how our results may inform future sampling strategies.

## Methods

### Study site

Soil samples were collected during September 2005 (the rainy season) from an area of disused land measuring 237.5 m^2^ (23.75 m×10 m) situated to one side of road 231 in Amphoe Meung, at a distance of 8 km northeast of Sappasithiprasong Hospital, Ubon Ratchathani, northeast Thailand. This is a rural rice-growing region where road traffic is light and cattle range through the area. The soil type was sandy loam, and was wet under foot but not flooded. The vegetation was low-lying scrub and the area showed no signs of cultivation. The site included a single concrete electricity pole. A brick wall formed one boundary, running parallel to and distal from the road.

Five people undertook sampling over a 2-day period of intermittent rain. The site was initially divided into a grid system using string and wooden stakes, in which 5×20 spots were plotted 2.5 m apart on the vertical axis, and 1.25 m apart on the horizontal axis. The grid was referenced alphabetically (A to E for horizontal rows as viewed back against the wall, row E lying closest to road) and numerically (1 to 20, moving across from left to right on vertical axis). Each point is hereafter termed a ‘sampling point’ and the specific site defined by its grid reference.

### Soil sampling

A hole was dug with a clean spade to a depth of approximately 30 centimetres. A clean plastic bag was placed on weighing scales and a sample of soil (100 grams) was removed from the base of the hole and placed into the bag. Each soil sample was labelled using pre-prepared stickers denoting the grid reference number. The bag was closed and stored out of direct sunlight at ambient temperature until transported to the laboratory where it was processed on the same day. The utensils used for sampling were cleaned between each use by rinsing with bottled water to remove visible debris, followed by cleaning with 70% ethanol and air drying.

### Soil culture and *B. pseudomallei* identification

Soil samples were batch processed at the end of each collection day. 100 ml of sterile water was added to each bag, mixed well and left overnight to sediment. The upper layer of water was then transferred by plastic pipette to a sterile plastic container. Four aliquots of 100 µl were spread plated onto each of 4 Ashdown's selective agar plates. A further 1 ml of the soil water sample was added to 9 ml of selective enrichment broth consisting of threonine-basal salt plus colistin (TBSS-C50 broth). This was incubated at 40°C in air for 48 h, after which 10 µl of surface liquid was plated onto a second Ashdown's agar plate which was incubated and observed as before. Agar plates were incubated at 40°C in air and visually inspected daily for 4 days. Colonies of *B. pseudomallei* were initially identified on the basis of colony morphotype. This included the characteristic colony morphology (purple, flat, dry and wrinkled) together with 6 additional colony morphotypes, as described previously [24]. Colonies suspected to be *B. pseudomallei* were tested using the oxidase test, and positive colonies confirmed as *B. pseudomallei* using a highly specific latex agglutination test (positive for *B. pseudomallei* but negative for *B. thailandensis*) [25],[26].

### Genotyping of *B. pseudomallei*

Genotyping of *B. pseudomallei* was performed for 3 sampling points (grid reference A11, D10 and E4). These were selected at random from sampling points that gave at least 200 *B. pseudomallei* colonies on the two primary Ashdown's agar plates. For each sample, 200 primary colonies were picked to purity and subjected to PFGE using *Spe*I, as previously described [27]. Analysis of PFGE banding patterns for the 200 colonies at each of the three sampling points was performed using the BioNumerics software version 2.5 (Applied Maths, Belgium). For the purposes of this study, interpretation was defined so as to be highly discriminatory. Isolates with identical PFGE banding patterns were regarded as genotypically indistinguishable, but isolates with one or more bands different were defined as putatively different and given a different banding pattern number. One bacterial representative of each banding pattern type was further examined using MLST, as previously described [23]. The alleles at each of the loci were assigned and sequence type defined using the *B. pseudomallei* MLST website (http://bpseudomallei.mlst.net).

### Measures of genetic diversity

Genetic diversity of *B. pseudomallei* within a given sampling point was defined using Simpson's index of diversity. This describes the probability that two randomly selected bacterial cells within a sampling point will be different genotypes; 0 indicates no diversity (all cells identical) and 1 indicates maximum diversity (all cells different). Confidence intervals for Simpson's index were calculated as described previously [28]. Further analysis was performed to examine whether the genetic distance between isolates within a given sample was significantly different from that expected if all isolates were randomly distributed between the three sites. The number of different alleles was determined for all 19,900 pairwise comparisons of the 200 colonies (strains) at each of the three sampling points. The results were compared to mean values calculated from 100 random samples, each of 200 strains, drawn with replacement from the combined data set of 600 strains (all 3 sampling sites). Statistical significance was gauged by calculating the 0.01, 0.05 and 0.95 and 0.99 percentiles from the re-sampled data. Genetic diversity between two sampling points was measured using the Morisita index of similarity. This ranges from 0 to 1; 0 indicates that no genotypes are shared between the two sampling points, and 1 indicates complete identity. All analyses were carried out using Stata 9.0 (College Station, Texas, United States), except the random resampling procedure which used a PERL script written by EJF (available on request).

## Results

**Figure 1:**
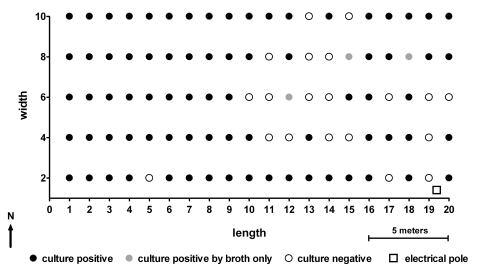
The presence of *B. pseudomallei* in 100 spaced sampling points within an area of disused land in northeast Thailand.

A total of 80 out of the 100 sampling points were culture positive for *B. pseudomallei*, of which 77 were positive from both direct plating onto Ashdown's agar and selective enrichment broth, and 3 were positive from selective enrichment broth culture alone (Figure 1). *B. thailandensis* was not detected. The genetic variability of *B. pseudomallei* was defined and compared within and between sampling points by genotyping 200 colonies at each of three positive points (A11, D10 and E4, see Figure 1). PFGE of 600 individual primary colonies revealed 12 PFGE banding pattern types. MLST of a single random isolate of each of the 12 PFGE types revealed 9 distinct sequence types (STs) (Table 1).

**Table 1:** Relationship between PFGE and MLST analysis of soil isolates.

PFGE Type	Sequence Type	MLST Profile						
		ace	gltB	gmhD	lepA	lipA	narK	ndh
1	ST 424	4	12	10	4	8	3	1
2	ST 177	1	1	4	3	1	3	1
3	ST 176	3	1	4	1	1	3	1
4	ST 185	1	4	2	2	1	4	1
5	ST 33	1	4	12	1	1	2	1
6, 11, 12	ST 60	3	1	12	1	1	3	1
7	ST 163	3	2	2	1	1	4	1
8, 10	ST 93	1	1	2	1	1	4	1
9	ST 304	1	1	5	1	1	4	1

Table 2 shows the breakdown of STs within each of the three sampling points. D10 and E4 each contained four STs and A11 contained three STs. Although the distance between the 3 sampling points was low (7.6, 7.9 and 13.3 meters for A11-D10, D10-E4, and A11-E4, respectively), only two STs were present in more than one sampling point (E4/D10; ST176, and D10/A11; ST60); no STs were detected in all three points, and no STs were common to E4 and A11 which were the two sites separated by the greatest distance. This strong segregation of STs was reflected in low Morisita index values (Table 2). Furthermore, each site was characterized by the following predominant genotypes, each of which was restricted to a single site: ST93 in A11 (87%), ST163 in D10 (51.5%), and ST185 in E4 (70%). Simpson's index of diversity ranged from 0.24–0.65 (Table 2).

**Table 2:** Genotyping results for 200 colonies of *B. pseudomallei* from each of three independent sampling points.

Sequence Type	Sampling Points		
	E4	D10	A11
ST 424	38 (19%)		
ST 177	12 (6%)		
ST 176	10 (5%)	29 (14.5%)	
ST 185	140 (70%)		
ST 33		50 (25%)	
ST 60		18 (9%)	9 (4.5%)
ST 163		103 (51.5%)	
ST 93			174 (87%)
ST 304			17 (8.5%)
Simpson Index of diversity (95% CI)	0.47 (0.40–0.54)	0.65 (0.60–0.69)	0.24 (0.16–0.31)
Morisita Index of similarity			
- compared to E4	-	0.02	0.00
- compared to D10	-	-	0.01

**Figure 2:**
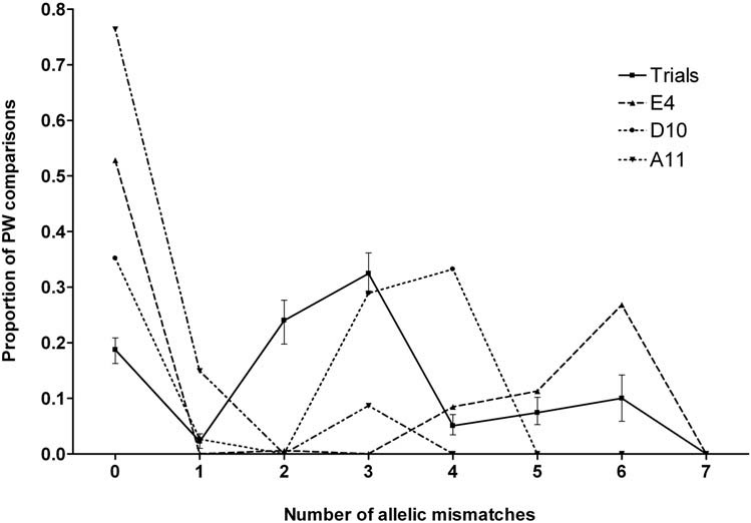
Graph of the proportion of all pairwise comparisons showing 0, 1, 2 … 7 allelic mismatches for each of 200 primary colonies (strains) examined at three independent sampling points. The “trials” data represents mean values for 100 random samples of 200 strains drawn from the combined data set of 600 strains from all three sampling points (with replacement). Error bars are based on the 5^th^ and 95^th^ percentiles of the 100 random samples. No pw comparisons (real or trial data) differ at all seven loci since the locus *ndh* is monomorphic (invariant).

The finding of very limited overlap between the sampling points was further examined by comparing the average pairwise distance (in terms of allelic mismatches) between isolates within each point to that expected if the three points were combined as a single population. Figure 2 shows the proportion of all 19,900 ((200*199)/2) pairwise comparisons showing 0, 1, 2 … 7 allelic mismatches for each of the seven MLST loci for 200 colonies (strains) examined at each of three independent sampling points. This was compared to randomized data derived from the mean values for 100 random samples of 200 strains resampled with replacement from the combined data set of 600 strains from all three points. All three sampling points showed a significantly greater proportion of identical pairs (i.e. the same ST) than the randomized data (P<0.01), which is consistent with localised clonal expansion. For two of the three sampling sites, no significant difference was observed in the proportion of related but non-identical STs (i.e. those differing at a single locus) compared with the randomized data. The exception was A11 where a significantly higher number of pairwise comparisons (P<0.01) corresponded to a single MLST locus difference; ST304 is a single locus variant of the predominant clone ST93, being variant at *gmhD*. Comparison of the two *gmhD* alleles (allele 2 for ST93 and allele 5 for ST304) indicated two base differences (C→T position 118, and T→C position 327). The third genotype noted at A11 is ST60; this differs at three loci from both ST93 and ST304, thus accounting for the small peak at three mismatches in the A11 plot.

Comparison of PFGE and MLST results demonstrated that two STs contained strains with variable PFGE banding pattern types. ST93 contained strains with two PFGE types (types 8 and 10), which were 12 bands different. This ST was only found in sampling point A11, in which the proportion of each banding pattern was 172/174 (99%) for type 8, and 2/174 (1%) for type 10. ST60 contained strains with three PFGE types (types 6, 11 and 12). The difference in banding patterns between these three was 6 bands (PFGE type 6 versus 11), 1 band (PFGE type 6 versus 12), and 2 bands (PFGE type 11 versus 12). All 18 ST60 isolates from D10 corresponded to type 6, whereas 8/9 ST60 isolates from A11 corresponded to type 11, and 1/9 to type 12. These PFGE data confirm fine-scale geographic structuring, even within a single MLST-defined clone. We also note differences between the single locus variants ST 304 (type 9) and ST93 (types 8 and 10); PFGE type 9 differed from type 8 by 18 bands, and from type 10 by 10 bands.

## Discussion

This study has demonstrated marked geographic structuring of *B. pseudomallei* genotypes in soil. The dramatic differences in genotype frequency over such small distances are striking, but difficult to interpret. One explanation is that the numerically dominant ST at each sampling point represents a strain with superior biological fitness compared with STs present as a minority of the population. This could relate to factors such as soil type or pH, or competition with other microbial species. This would assume that adjacent foci of soil have variable microenvironments, but it seems unlikely that nearby sampling points within a confined area of disused land would differ sufficiently to support multiple, non-overlapping niches. An alternative possibility is that of local competition between clones of *B. pseudomallei*. Flooding or other disturbance mechanisms would provide the means for a given clone to migrate and become established within a specific plot. Once the clone has reached a certain threshold frequency, it could repel invaders either by the production of microbicides, through phage to which they themselves are resistant, or via other killing mechanisms. The presence of a clone as a minority population could represent the ability of this strain to survive at a lower level, or could represent the boundary of a point of predominance in an adjacent point or focus.

This study also provides evidence for microevolution of *B. pseudomallei* in soil. PFGE is a more sensitive marker of very rapid genetic change than MLST in this species. Alterations in banding pattern arise due to any kind of genetic event that alters the presence or absence of restriction sites anywhere in the genome, or else changes the distance between existing sites. In contrast, MLST genes are chosen specifically to code for a central housekeeping role and to be highly conserved. MLST is therefore blind to large-scale genomic rearrangements that may dramatically alter the PFGE banding pattern [29]. Two of the nine STs contained strains with variable PFGE banding pattern types. We postulate that these changes represent microevolution within our sampling site rather than importation of several strains with matching ST but a different banding pattern. This is consistent with the finding that genomic islands constitute ∼6% of the *B. pseudomallei* K96243 genome [30]. Furthermore, comparison of the whole genome sequences of *B. pseudomallei* and *B. mallei* indicated the capacity for genomic rearrangement and gene loss by two species that are highly related by MLST [31]. Our findings are consistent with a dynamic genome that is evolving through the movement of genomic islands and rearrangements such as inversions and indels.

The co-existence in a single soil sample of a single locus variant of ST93 (ST304) can be explained by *in situ* microevolution or by a chance association. ST93 and ST304 have both been isolated previously in northeast Thailand. We recovered ST93 from the environment in 1990, 1998 and 1999, each from different sampling sites situated along road 212 which runs northwest from the town of Ubon Ratchathani. The MLST database (www.mlst.net) contains a fourth ST93 isolate that was associated with human disease in Thailand in 1998. We have also recovered ST304 from two patients with melioidosis presenting to a hospital in northeast Thailand in 1999. However, an accurate picture of the distribution and frequency of co-localization in soil of ST93 and ST304 in this region has not been defined, and it is difficult to speculate on the probability of a chance association.

A potential pitfall of this study is that the proportion of each ST was obtained after the soil sample had been prepared by mixing with water and overnight sedimentation followed by growth using rich media. Some STs may be more adapted to survival or growth after the addition of distilled water during sample preparation, or may move more efficiently into the layer of surface water that is removed for culture. It is also possible that some STs are more likely to grow on laboratory media than others, and that some STs are viable but non-culturable under the conditions used. Resolution of these issues will require the direct application of molecular tools to soil samples, and comparison of genotypes with those obtained using conventional culture and existing soil preparation methods.

Our findings have several important implications for future genotyping studies. Soil sampling at a single location will fail to identify the genotypes present at a distance of even a few meters. Furthermore, the predominance of a single ST at a given site requires that extra sampling effort is required to detect any genotypes present as a minority of the population. We estimate that the characterization of approximately 50 colonies from any single site would provide an 85% probability of detecting a genotype present at the site at a frequency of 2%. This is based on the exact 95% binomial confidence interval for ST60 at sampling point A11, which was present in the lowest proportion. The temporal stability of the genotype distribution described here is not known, and it is possible that markedly different genotypic frequencies might be recorded from the same sampling sites if the study were to be repeated at some point in the future. It is also unclear whether the degree of genetic diversity described here will be reproduced within Thailand and in other endemic countries, although a study by Pearson et al. in which genetic diversity was demonstrated by variable number tandem repeat (VNTR) analysis within a small geographic area of Australia [32] suggests that this will prove to be a reproducible finding. The basis on which PCR is used in future studies to detect *B. pseudomallei* in the environment also requires careful consideration. Amplification may give rise to mixed products, and DNA from strains present at low copy number may go undetected. PCR may become an appropriate technology for the detection of *B. pseudomallei*, but is not an appropriate basis for subsequent genotyping unless multiple independent amplicons are evaluated.

This study has investigated *B. pseudomallei* in soil taken from an area of disused land. This is in contrast to many previous studies in Thailand which were conducted in rice paddies. The basis for our choice was to examine an environment free of external influences such as chemical fertilizers and pesticides, together with the effect of ploughing, planting, burning of rice stubble and the presence of rice plants. However, most disease is probably acquired in rice paddies; further studies are underway to compare and contrast the findings reported here with those from a rice paddy in the same region.
